# Biodegradable Polymeric Nanoparticles Loaded with Flavonoids: A Promising Therapy for Inflammatory Bowel Disease

**DOI:** 10.3390/ijms24054454

**Published:** 2023-02-23

**Authors:** Mingrui Li, Ying Liu, Benno Weigmann

**Affiliations:** 1 Department of Medicine 1, Kussmaul Campus for Medical Research, University of Erlangen-Nürnberg, 91052 Erlangen, Germany; 2 Medical Immunology Campus Erlangen, Friedrich-Alexander University Erlangen-Nürnberg, 91052 Erlangen, Germany

**Keywords:** inflammatory bowel disease, flavonoids, biodegradable, nanoparticles, polymers, synergy

## Abstract

Inflammatory bowel disease (IBD) is a group of disorders that cause chronic non-specific inflammation in the gastrointestinal (GI) tract, primarily affecting the ileum and colon. The incidence of IBD has risen sharply in recent years. Despite continuous research efforts over the past decades, the aetiology of IBD is still not fully understood and only a limited number of drugs are available for its treatment. Flavonoids, a ubiquitous class of natural chemicals found in plants, have been widely used in the prevention and treatment of IBD. However, their therapeutic efficacy is unsatisfactory due to poor solubility, instability, rapid metabolism, and rapid systemic elimination. With the development of nanomedicine, nanocarriers can efficiently encapsulate various flavonoids and subsequently form nanoparticles (NPs), which greatly improves the stability and bioavailability of flavonoids. Recently, progress has also been made in the methodology of biodegradable polymers that can be used to fabricate NPs. As a result, NPs can significantly enhance the preventive or therapeutic effects of flavonoids on IBD. In this review, we aim to evaluate the therapeutic effect of flavonoid NPs on IBD. Furthermore, we discuss possible challenges and future perspectives.

## 1. Introduction

Inflammatory bowel disease (IBD), which includes Crohn’s disease (CD), ulcerative colitis (UC), and IBD unclassified (IBDU), is a group of chronic or relapsing inflammatory diseases that primarily affect the small and large intestine [1]. In general, abdominal pain, unexplained fever, fatigue, diarrhoea, and loss of appetite as well as weight loss are the most common symptoms of IBD [2], of which UC is characterised by uninterrupted inflammation occurring mainly in the colon, whereas CD can occur as focal inflammation throughout the gastrointestinal tract (GI). In addition, inflammation in UC occurs only in the colonic mucosa. In contrast, CD can involve multiple layers of the bowel wall [3]. Currently, more than 4.9 million people in Western society suffer from IBD [4]. In addition, the prevalence of IBD in Asia has increased markedly, imposing a huge economic burden on individuals and societies [5].

**Figure 1:**
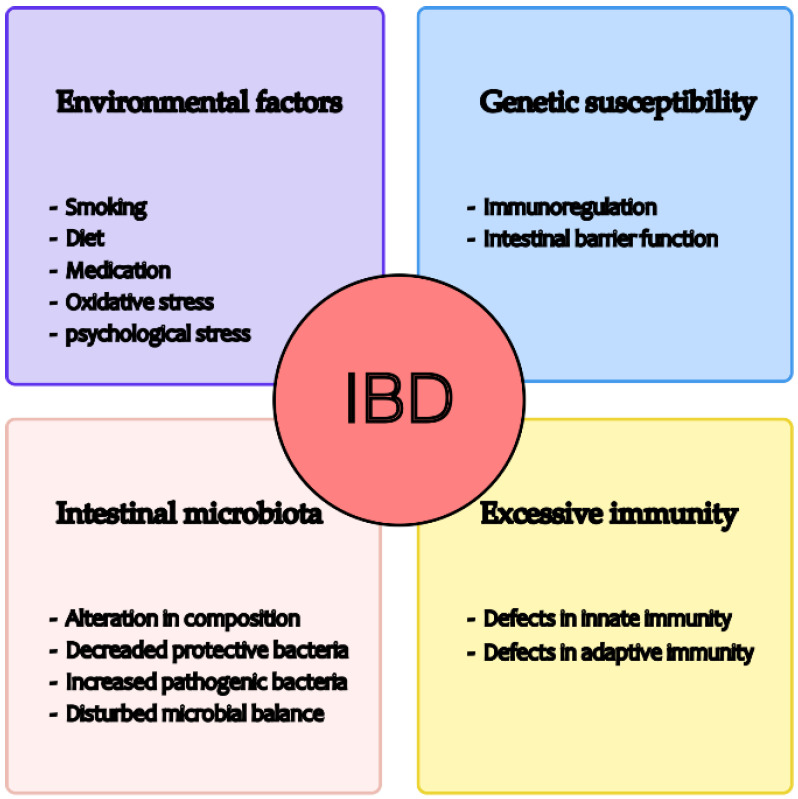
Possible causes of IBD development.

Recent studies have shown that the pathogenesis of IBD is related to environmental factors, gut microbiota, excess immunity, and genetic susceptibility (see Figure 1) [6]. With regard to the environment, studies have shown that a diet rich in fruit and vegetables is associated with a reduced risk of Crohn’s disease [7], while the consumption of fast foods high in sugar and fat may worsen the onset of the disease [8]. In addition, smoking, medication, and oxidative and psychological stress may influence the development of IBD [9,10,11]. Second, some studies have shown that the gut microbiota of IBD patients is significantly different from that of healthy individuals, such as altered composition, decreased protective bacteria, increased pathogenic bacteria, and disturbed microbial balance. These changes, in turn, may influence the progression of IBD [12,13,14]. Thirdly, the intestinal immune system comprises innate and adaptive immunity. Innate immunity includes various aspects such as the protective barrier function of the intestinal mucosa, antibacterial proteins, gastric acid and immune cells, and innate cytokines and molecules. On the other hand, adaptive immunity, involving T and B cells, is pathogen-specific and is typically triggered when the innate immune system fails to respond effectively to a pathogen [15]. Regulation of the immune response to the gut microbiota is critical in maintaining the balance between immune tolerance and defensive inflammation, and any disruption can lead to excessive immunity, triggering the onset of IBD [16]. Genetic susceptibility has been demonstrated in IBD patients [17]. Investigation of the genes and genetic loci involved in IBD has shown that several key processes are critical for maintaining intestinal stability, such as epithelial barrier function, immune regulation, and so on. A disrupted epithelial barrier can allow microbial invasion, which is then recognised by the innate immune system, leading to the initiation of appropriate responses, such as tolerogenic, inflammatory, and restorative responses, in part through the secretion of extracellular mediators that attract T and B cells [18]. Nevertheless, the precise cause of IBD remains unclear [6]. 

Polyphenols are a group of phytochemicals found in the vegetable kingdom. Recent studies have shown that certain polyphenols are able to prevent or alleviate symptoms of IBD through multiple pathways [19,20,21,22]. Being a subclass of polyphenols, flavonoids have a specific type of molecular structure, which makes them a research hotspot in the current alternative therapy field of IBD. Flavonoids are thought to have beneficial effects on the human body, such as inhibiting oxidative reactions, reducing inflammatory effects, and reducing the risk of chronic diseases [23,24]. In recent years, a number of studies have documented that some types of flavonoids, such as quercetin, apigenin, naringenin, and epigallocatechin-3-gallate (EGCG), have the ability to protect against IBD [25,26,27,28]. For example, in a double-blind, placebo-controlled pilot study, Dryden et al. found that EGCG significantly reduced disease activity index scores in patients with mild to moderate UC. In addition, oral administration (400 mg/800 mg EGCG daily) for two months caused only minor adverse effects [29]. Nevertheless, the use of flavonoids in IBD is still unsatisfactory. The bioavailability of flavonoids is low due to poor absorption, rapid metabolism, and other reasons [30], resulting in low bioactivity in vivo and severely limiting their clinical use in the treatment of IBD. In order to improve the bioactivity of flavonoids in vivo, many attempts have been made to increase bioavailability, which is considered to be a determinant factor of bioactivity in vivo. Currently, with the development of nanomedicine, a nanodelivery system has been used to enhance the bioavailability of flavonoids. Nanocarriers can efficiently encapsulate various flavonoids to form nanoparticles (NPs), which greatly enhance the bioavailability of flavonoids. In particular, novel biodegradable polymers have been developed significantly in the last decade and can be used to form NPs. In conclusion, the preventive and therapeutic functions of flavonoids against IBD can be significantly enhanced by biodegradable polymeric NPs.

In this review, we introduce different types of NPs and focus on the application of biodegradable polymeric nanoparticles in the delivery of flavonoids. We also discuss the future challenges and prospects of flavonoid NPs.

## 2. Flavonoids 

### 2.1. Classification, Metabolism and Absorption of Flavonoids

**Figure 2:**
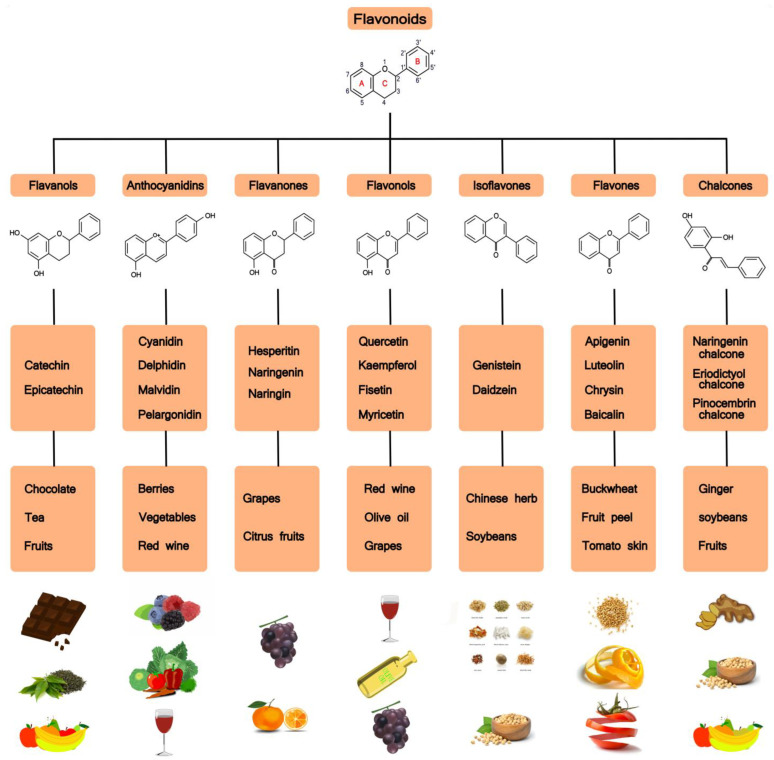
Flavonoids: chemical structures, subclasses, and food sources.

Flavonoids are a large family of polyphenolic plant compounds. Flavonoids have a three-ring basic structure consisting of two benzene rings linked by a pyran heterocyclic ring. Based on structural differences, flavonoids are mainly divided into seven subsets, namely flavonols, flavanones, isoflavones, anthocyanidins, flavanols, flavones, and chalcones (see Figure 2). To date, scientists have discovered over 5000 different flavonoids from different plants [31]. 

Aglycones, glycosides, and methylated derivatives are the main forms of flavonoids in vivo, with aglycones being the basic structure of flavonoids [32]. In contrast, most flavonoids in nature occur in the form of glycosides [30]. The absorption of flavonoids in vivo is largely dependent on their physicochemical properties (mainly molecular weight, solubility, configuration, lipophilicity, pKa) [32]. Due to their hydrophilic nature, glycosides hardly cross the cell membrane in the intestine. Consequently, glycosides can only be absorbed in the intestine through the hydrolytic action of sugar conjugates or a specific active transport pathway [33]. In the small intestine, where glycosides cannot be absorbed, aglycones can pass through epithelial cells by passive diffusion and reach the colon directly, where they are subsequently converted into aglycones by colonic microorganisms. In the colon, unabsorbed aglycones are simultaneously degraded by microbiota into small molecule compositions that are further degraded or absorbed [30,32,33]. In addition, some flavonoid aglycones are reabsorbed into the small intestine via bile secretion. Due to specific conjugation reactions, the free forms of aglycones do not exist in the blood or urine, except for catechins [34]. In addition, metabolism can take place in the liver and kidneys via the bloodstream [35].

### 2.2. Mechanisms of Flavonoids Regulating IBD

**Figure 3:**
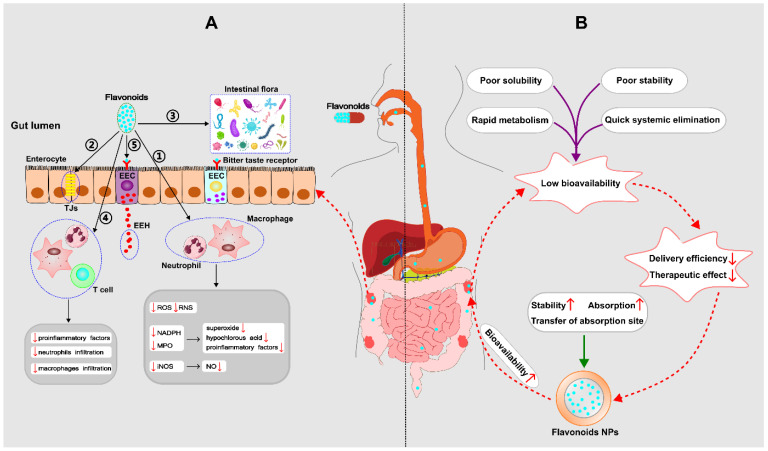
(**A**) Mechanisms by which flavonoids regulate IBD––① Antioxidant property; ② Preservation of the impaired epithelial barrier: flavonoids have the ability to increase mucus, improve tight junctions (TJs) and the ultrastructure of the intestinal mucosa; ③ Shaping of the intestinal microbiota: Flavonoids are able to restore the damaged microbial diversity, increase the number of anti-inflammatory bacteria, and inhibit the translocation of harmful bacteria; ④ Immunomodulatory function; ⑤ Modulation of the enteroendocrine system: flavonoids can stimulate the synthesis and release of enteroendocrine hormones (EEH) resulting from enteroendocrine cell (EEC), which produces a beneficial effect on IBD. (**B**) Mechanisms of nanoparticles (NPs) to improve the bioavailability of flavonoids. (ROS: reactive oxygen species; RNS: reactive nitrogen species; NADPH: nicotinamide adenine dinucleotide phosphate; MPO: myeloperoxidase; iNOS: inducible nitric oxide synthase; NO: nitric oxide; 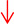 down; 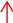 up).

**Figure 4:**
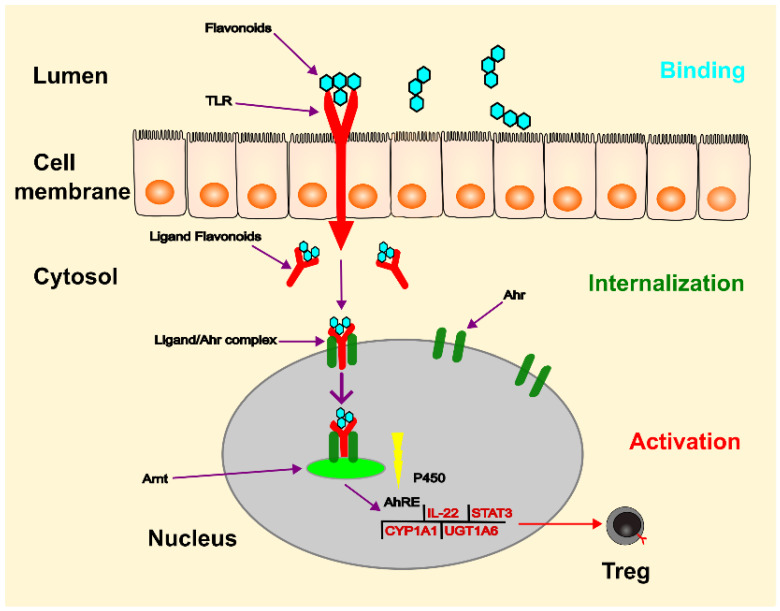
The process of gene regulation by Ahr can be simplified as follows: flavonoids bind to TLR in the lumen and are then internalised into the cytosol. This complex acts as a ligand for Ahr, leading to the release of associated proteins and translocation of the complex to the nucleus. Ahr then dimerises with Arnt and the Ahr/Arnt complex binds to the AhRE, triggering transcription of target genes. This process leads to increased production of IL-22 and cytochrome P-450, and decreased production of STAT3 and NF-κB. The ligands can also act on the cytoplasm by altering the function of various proteins through a cascade of protein phosphorylation facilitated by AhR-associated protein kinases. (Ahr: aryl hydrocarbon receptor; TLR: toll-like receptor; Arnt: Ahr nuclear translocator; Ah: aryl hydrocarbon; AhRE: Ah response element; CYP1A1: cytochrome P450 family 1 subfamily member 1; UGT1A6: UGT-glucuronosyltransferase family 1 member A6).

In 1976, Galsanov et al. first discovered that quercetin, a member of the flavonoid family, could be used to reduce intestinal inflammation [36]. Subsequently, other flavonoids were identified as anti-inflammatory phytochemicals. To date, several pathways of flavonoid regulation of IBD have been documented, including antioxidant activity, preservation of the intestinal epithelial barrier, shaping of the intestinal microbiota, immunomodulatory function in the intestine, and modulation of the enteroendocrine system [37] (see Figure 3A). For the detailed molecular mechanisms, see also Figure 4.

#### 2.2.1. Antioxidant Property

Over the past few decades, reactive nitrogen species (RNS) and reactive oxygen species (ROS) have been identified as critical participants in the pathogenesis of IBD [38]. Recent studies have shown that excessive production of RNS and ROS is present in the inflamed intestine [39]. On the one hand, in patients with IBD, excessive mononuclear cells and neutrophils accumulate in the inflamed tissue of the intestine. Subsequently, myeloperoxidase (MPO) activity increases and the nicotinamide adenine dinucleotide phosphate (NADPH) oxidase system is activated, leading to the overproduction of superoxide and hypochlorous acid. Thus, cytotoxicity directly attacks the affected intestinal tissue [40]. In addition, the above pathway also produces a large amount of multiple pro-inflammatory factors [41]. Nevertheless, some flavonoids can significantly reduce MPO activity in the colon [42]. In addition, flavonoids can inhibit the overproduction of RNS-ROS in colitis models [43]. On the other hand, under physiological conditions, a small amount of nitric oxide (NO) usually directly protects against inflammatory injury. However, in the inflammatory state, a large amount of NO is produced due to the overexpression of inducible nitric oxide synthase (iNOS). Subsequently, through the action of superoxide anions, excessive NO causes overproduction of peroxynitrites, leading to intestinal destruction [44]. Recent research has shown that flavonoids can inhibit the expression of iNOS, resulting in an apparent reduction in NO production [45]. For the above reasons, flavonoids are able to reduce the enterotoxicity induced by NO.

#### 2.2.2. Preservation of Epithelial Barrier

The intestinal epithelial barrier is an interface between the environment and the host tissue that plays an essential role in maintaining intestinal homeostasis [46]. At present, it remains controversial whether the loss of the epithelial barrier is a major cause or a consequence of IBD. However, an integral intestinal epithelial barrier is critical to prevent the development of IBD. Conversely, loss of the epithelial barrier marks the progression and duration of IBD [47]. Therefore, improving the epithelial barrier may improve the outcome and prognosis of IBD. Recent studies have shown that flavonoids benefit the integrity of the intestinal epithelial barrier. Bian et al. investigated the protective effect of kaempferol on barrier dysfunction in a coculture model of Caco-2 cells and endothelial cells and found that kaempferol attenuated the decrease in transepithelial electrical resistance (TEER) and the overproduction of interleukin-8 (IL-8) induced by lipopolysaccharide (LPS). Furthermore, kaempferol alleviated the LPS-induced decrease in tight junction protein expression and reduced the protein expressions of nuclear factor-κB (NF-κB) phosphorylation level and inhibitor of NF-κB (I-κB) induced by LPS [48]. Wu et al. established murine colitis models induced by dextran sodium sulphate (DSS) and investigated the protective effect of oral or rectal EGCG administration on experimental colitis and found that oral EGCG increased colonic mucus, protected tight junctions, and colonic mucosal ultrastructure [28].

#### 2.2.3. Shaping Microbiota 

Gut microbiota dysbiosis is closely associated with the pathogenesis of IBD, which is characterised by reduced biodiversity, decreased stability, and an increase in Proteobacteria (mainly Bilophila, Enterobacteriaceae and some units of Bacteroidetes) [49]. In turn, dysbiosis may lead to intestinal inflammation by affecting the host immune system or some metabolic pathways [50]. Recent studies have shown that dietary flavonoids have the potential to protect against IBD by regulating intestinal dysbiosis [51]. For example, Ren et al. found that acacetin alleviated intestinal flora dysbiosis in rodents with colitis, which was characterised by decreased Firmicutes and increased Escherichia-Shigella, which may cause the overproduction of inflammatory cytokines in IBD [52]. Mu et al. investigated the correlation between bacteria regulated by anthocyanins and anthocyanin-induced anti-inflammatory effects by establishing DSS-induced colitis in mice and found that anthocyanins inhibited the decrease of Bifidobacterium and Lactobacillus and prevented the increase of Gammaproteobacteria and Helicobacter after DSS treatment. Furthermore, Mu et al. established non-pathogenic-dependent and pathogenic-dependent microenvironments after treatment with broad-spectrum antibiotics. They found that both oral administration of anthocyanins and non-pathogenic treatment altered the expression of tight junction proteins in the colon and preserved colonic architecture. However, the non-pathogenic treatment didn’t alleviate intestinal inflammation compared to the oral administration of anthocyanins. In addition, the pathogen-dependent dysbiosis was exacerbated by the increase of pathogens (such as Helicobacter) in the colon. Based on the above results, the author concluded that anthocyanins can maintain intestinal homeostasis in DSS-induced colitis mice by regulating dysbiosis in the colon, which may help to alleviate intestinal inflammation [53].

#### 2.2.4. Immunomodulatory Function

An abnormality of the intestinal immune system can lead to IBD, which includes the innate immune system (mainly dendritic cells, macrophages, and neutrophils) and the adoptive immune system (T lymphocytes and B lymphocytes). Among these immune cells, T lymphocytes play an important role in the pathogenesis of IBD, which is characterised by specific cytokine responses [3,54]. Wu et al. discovered that EGCG induced apoptosis of CD4(+) T cells by activating signal transducer and activator of transcription 1 (STAT1) [55]. Tao et al. found that icariin, a natural flavonoid glucoside, promoted amelioration of experimental murine colitis by suppressing Th1/Th2 overreactions leading to overproduction of proinflammatory cytokines [56]. Similarly, macrophages are involved in the pathogenesis of IBD and produce excessive pro-inflammatory factors. Some in vivo studies have shown that flavonoids have an anti-inflammatory function by suppressing macrophage activity. For example, Camuesco et al. established murine colitis models induced by DSS and found that quercitrin significantly inhibited macrophage infiltration in the affected colon [45]. In addition, neutrophil infiltration normally occurs in the inflamed bowel of IBD patients. There is growing evidence that some flavonoids have the potential to alleviate IBD by suppressing neutrophil infiltration in the inflamed colon [57].

An abnormal response by intestinal immune cells can facilitate the synthesis and release of pro-inflammatory cytokines such as interleukin-1 beta (IL-1 beta), interleukin-6 (IL-6), interferon-gamma (IFN-gamma), and tumour necrosis factor (TNF-alpha). These cytokines play a critical role between an abnormal immune response and IBD. Their production may be linked to inhibition of mitogen-activated protein kinase (MAPK), NF-κB, and STAT activation. Recent studies have shown that some flavonoids can block the above pathways, thereby reducing the inflammatory level of IBD [58,59,60,61].

#### 2.2.5. The Modulation of Enteroendocrine System

Recently, Li et al. reported that flavonoids may protect against IBD by regulating the enteroendocrine system [37]. In particular, a large number of studies have shown that flavonoids can stimulate the secretion of some enteroendocrine hormones, such as cholecystokinin (CCK), ghrelin, glucagon-like peptide (GLP-1), and glucagon-like peptide (GLP-2) [62,63,64,65,66,67,68]. In addition, GLP-1/2 have great potential in the treatment of IBD. The latent protective mechanisms probably include GLP-1/2 and have the ability to promote the improvement of the impaired epithelial barrier; GLP-1/2 are able to regulate the differentiation of T lymphocytes and their functions; and the innate immune cells (macrophages, dendritic cells, etc.) could be regulated by GLP-1/2 [69,70,71,72,73,74]. Ghrelin has been reported to reduce the degree of intestinal inflammation in colitis-infected mice [75,76,77]. CCK also has an anti-inflammatory effect, as shown by some in vivo and in vitro studies [78,79,80,81]. In conclusion, flavonoids exert indirect therapeutic effects on IBD through direct modulation of the enteroendocrine system.

#### 2.2.6. Molecular Mechanisms of Flavonoids Modulating IBD

The aryl hydrocarbon receptor (Ahr) is located primarily in lymphocytes and dendritic cells of the intestinal mucosa and plays a role as an intracellular transcription factor for the actions of flavonoids [82] (see Figure 4). During digestion in the gastrointestinal tract, flavonoids are broken down into smaller molecules. Once these molecules enter the intestinal lumen, they bind to the toll-like receptor (TLR) on the plasma membrane and are transferred to the cytosol. As a ligand, they then bind to the Ahr, which triggers its translocation to the nucleus. Through dimerisation with the Ahr nuclear translocator (Arnt) protein, the flavonoid-Ahr-Arnt complex binds to the Ah response element (AhRE) located in the 5V flanking region of various genes, activating the target genes. These genes can regulate the expression of cytochrome P-450, regulatory T cells, STAT3, and interleukin-22 (IL-22), of which IL-22 is crucial for maintaining intestinal integrity and increasing the production of mucus and beta-defensin-2 [83]. Flavonoids act as important immunomodulators, inducing a beneficial pattern of cytokines and immune cells that counterbalance the inflammatory changes in the intestinal mucosa [82]. 

In addition, some studies have shown that flavonoids bind to bitter taste receptors expressed in enteroendocrine cells and induce gut hormone secretion [84,85,86]. By modulating gut hormones, flavonoids have a beneficial effect on IBD. For those interested in understanding the detailed molecular mechanism of enteroendocrine regulation, please read the review by Li [37].

### 2.3. Bioavailability of Flavonoids

Although flavonoids have multiple protective pathways against IBD, the clinical use of flavonoids is unsatisfactory due to their low bioavailability. Bioavailability refers to “the rate and extent to which the active ingredient from a drug is absorbed and becomes available at the site of action”, which is crucial for bioactive compounds to be effective in organisms. The above principle also applies to flavonoids in IBD [30]. Therefore, the bioavailability of flavonoids is of paramount importance for their bioactivity in vivo. Unfortunately, the bioavailability of flavonoids in vivo is comparatively low and can be influenced by numerous factors, such as molecular weight, glycosylation, metabolic conversion, and interaction with colonic microbiota [30]. All of these confounding factors contribute to the poor solubility and stability, rapid metabolism, and systemic elimination that make flavonoids so poorly bioavailable. Accordingly, the delivery efficiency and therapeutic effect are obviously reduced. 

In order to improve the bioavailability of flavonoids in vivo, scientists focus on improving some metabolic processes related to bioavailability, including increasing intestinal absorption [87], enhancing metabolic stability [88], transferring absorption site [89], and so on. To achieve the above goals, nano-delivery systems have been applied to flavonoids. Recently, with the development of biodegradable polymers, flavonoid-loaded polymeric NPs have increasingly become the therapeutic option targeting IBD, which can promote stability and absorption and also change the absorption site (see Figure 3B).

## 3. Biodegradable Polymeric Nanoparticles

A nanoparticle (NP) is a particle of matter between 1 and 100 nanometres (nm) in diameter. The definition is occasionally applied to larger particles, up to 500 nm. It has been confirmed that an NP has the potential to improve the solubility and stability of an encapsulated drug, enhance transport across membranes, and prolong retention time to improve efficacy and safety [90,91]. Based on these advantages, NP research has been increasingly used for drug development and application. Currently, nanoparticles (NPs) are mainly divided into three classes based on structural differences, namely polymeric NPs, inorganic NPs and lipid-based NPs, each of which has its own strengths and weaknesses. For example, inorganic NPs are well suited for theranostic applications due to their unique electrical, magnetic, and optical properties. However, toxicity and solubility limitations have become the obstacle to clinical therapy for inorganic NPs. Lipid-based NPs have simple structures, high bioavailability, and payload flexibility, while their encapsulation efficiency (EE) is low. Compared with the above NPs, polymeric NPs have more advantages, such as high payload for hydrophilic and hydrophobic drugs, precise control of particle properties and easy surface modification, as well as high EE. In addition, they are stable during storage, especially biodegradable polymers with low aggregation and toxicity [92]. For these reasons, biodegradable polymeric NPs have increasingly become a focus of research in nanomedicine. In recent years, more and more studies on biodegradable polymeric NPs loaded with flavonoids have been published. Therefore, we will focus on the presentation of biodegradable polymeric NPs in the following part.

### 3.1. Formulations 

**Figure 5:**
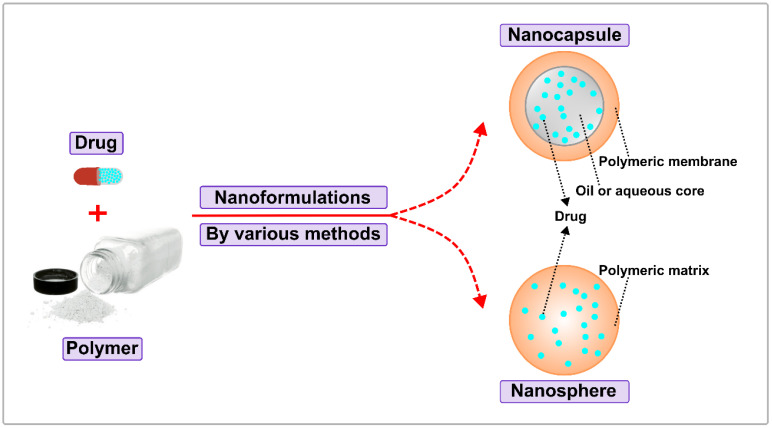
Classification of Biodegradable Polymeric Nanoparticles.

The most typical formulations of biodegradable polymeric nanoparticles contain nanocapsules and nanospheres. The structure of a nanocapsule is a cavity surrounded by a polymeric shell or membrane. In contrast, the nanosphere is an architecture of a solid matrix system (see Figure 5). Within the two main classes, biodegradable polymeric NPs are divided into configurations such as polymersomes, micelles, and dendrimers [92]. For example, a polymersome is a synthetic vesicle whose membrane is formed by amphiphilic block copolymers. Similar to liposomes, polymersomes are generally locally reactive, but have improved stability and drug retention efficiency. Because of these properties, the polymersome is able to effectively deliver the drug to the cytosol [93,94,95]. Polymeric micelles self-assemble to form a nanosphere with a hydrophilic core and hydrophobic shell, which is also a responsive block copolymer. These structural features are beneficial to protect aqueous drugs and prolong retention time. Dendrimer is a hyperbranched polymer that has intricate three-dimensional structures that allow for extensive control of size, shape, mass, and surface chemistry. Biodegradable polymeric NPs are formed by various methods including emulsification (solvent displacement/diffusion), nanoprecipitation, microfluidics, and ionic gelation [96,97,98,99,100]. In general, the type of polymers and encapsulated drugs determine the preferred NP formulation and synthesis method. When it comes to the type of polymers, biodegradable polymers are mainly divided into two main forms based on their sources, namely, natural biodegradable polymers and synthetic biodegradable polymers. Below, we will explain each of the two types of biodegradable polymers.

### 3.2. Natural Biodegradable Polymers

Natural biodegradable polymers are mainly derived from plants and animals. Due to their abundance in nature, biocompatibility, biodegradability, and low toxicity, these polymers are widely used in nano-delivery systems. However, they can cause immune rejection in vivo and are batch-to-batch variable, limiting their use in the clinic. Natural polymers consist mainly of protein-based polymers and polysaccharides. Protein-based polymers include zein, albumin, gelatin, soy, collagen, and silk fibroin. Polysaccharides include chitosan, dextran, agarose, hyaluronic acid, alginate, carrageenan, and cyclodextrin [101]. Among these polymers, chitosan, zein, alginate, and hyaluronic acid are more commonly used in nano-delivery systems and will therefore be highlighted (see Table 1). 

#### 3.2.1. Chitosan

Chitosan is a type of polysaccharide derived from chitin, which is abundant in the exoskeleton of crustaceans [102]. The backbone structure of chitosan contains 2-amino-2-deoxy-D-glucose and 2-acetamido-2-deoxy-D-glucose units bridged by β-(1-4) linkages. As one of the most widely used natural polymers for nanodelivery, chitosan has many advantages, such as combination with other polymers, easy surface modification, no toxicity, biocompatibility, biodegradability, anti-tumour/antioxidant/antibacterial properties, and, moreover, it is inexpensive and convenient to obtain [103]. Among these advantages, its surface modification and ability to bind with other polymers greatly increase the application value of chitosan. For example, by modification with folate, chitosan NPs loaded with 10-hydroxycamptothecin (HCPT) significantly improved tumour targeting and anti-tumour efficacy [104]. By mixing with poly(lactic-co-glycolic acid), namely, PLGA (a synthetic polymer), chitosan NPs loaded with catechin hydrate showed higher toxicity to cancer cells [105]. However, chitosan cannot be dissolved in water and is only soluble in acidic solutions, which limits its clinical value to some extent. 

#### 3.2.2. Zein

A natural polymer, zein is derived from maize and belongs to the prolamin family. It is also a combination of four components (α, β, γ, δ) with different molecular sizes, peptide chains, and solubilities. Zein is a hydrophobic protein, which means that it is poorly soluble in water but soluble in aqueous solutions containing ethanol, acetone, and other organic solvents [106]. Due to its hydrophobicity, biodegradability, biocompatibility, and safety, zein has been extensively used as a promising carrier for the nano-delivery of lipid-soluble chemicals. In drug delivery, zein can contribute to high bioavailability, controlled release, and drug protection/targeting [107]. For example, zein induced an 8200-fold increase in the water solubility of curcumin NPs compared to free curcumin due to the hydrophobic interaction between curcumin and zein. Furthermore, the chemical stability of curcumin NPs during storage was apparently improved [108]. Chen et al. reported that zein-hyaluronic acid-quercetin NPs possessed sufficient thermal stability and controlled release characteristics, accompanied by high encapsulation efficiency of the active flavonoid [109]. 

#### 3.2.3. Alginate

Alginate is a water soluble salt of alginic acid. As an anionic hydrophilic polysaccharide, alginate occurs naturally in all species of brown algae of the family Phaeophyta. The growth and seasonal conditions of the algae determine different structures of alginates. It is a linear natural polymer containing two uronic acids, namely, 1,4-linked β-D-mannuronic acid (M) and α-L-guluronic acid (G). These structural features make alginates anionic in nature due to the presence of carboxyl groups [110]. Alginate is acid-resistant, non-toxic, biocompatible, and mucoadhesive, which shows a gel-forming property in the presence of divalent cations such as Ca^2+^, Zn^2+^ [111]. Recent studies have reported that the combination of chitosan and alginate, used for targeted drug delivery, can apparently improve the therapeutic effect of NPs due to the universality of chitosan and the acid-resistant character of alginate [112]. In addition, as a hydrophilic carrier, alginate NPs can prolong the retention time of the drug and reduce the degradation of the drug in acidic environments due to the acid resistance. In addition, no aggregation of alginate particles is detected in critical organs [113].

#### 3.2.4. Hyaluronic Acid

As a natural adhesive polymer, hyaluronic acid (HA) is widely distributed in connective, epithelial, and neural tissues. HA has a high molecular weight and consists of two disaccharide units (D-glucuronic acid, N-acetylglucosamine). The natural biological effects of HA depend mainly on the length of the polysaccharide. HA has multiple physiological functions, including tissue growth, skin protection, bio-lubrication, modulation of water diffusion, maintenance of vascular elasticity, and promotion of wound healing. In addition, HA is biocompatible, biodegradable, and non-toxic. Currently, HA is widely used in nano-delivery systems. For example, a novel HA-based quercetin nanoformulation showed greater suppression of tumour growth compared to free quercetin [114].

### 3.3. Synthetic Biodegradable Polymers

Synthetic biodegradable polymers can be engineered for required properties, such as hydrophobicity, charge, and biodegradation profile. Based on different properties, these synthetic polymers are optimised for different drugs, delivery routes, and disease targets. Compared to natural polymers, synthetic materials are controlled for lower batch-to-batch variability, making them suitable for large-scale production. In addition, synthetic polymers have higher purity and are more homogeneous than natural polymers [115]. However, unintended products or metabolites during the degradation process may contribute to the cytotoxicity or immunogenicity of the synthesis. Currently, research on synthetic polymers in the field of nanomaterials is growing exponentially. As a result, a large number of synthetic biodegradable polymers have entered the market, such as polyester polylactide (PLA), poly(lactide-co-glycolide) (PLGA), poly(ε-caprolactone) (PCL), cyclodextrins (CDs), poly(β-amino esters) (PBAE), chain splitting polymer therapeutics (CSPTs), polyanhydride, dendrigraft poly-l-lysine (DGL), micelles, chitosan derivatives, and so on [113,116]. In the following sections, PLGA, PCL, DGL and polyanhydride will be explained in detail (see Table 1).

#### 3.3.1. Poly(Lactide-Co-Glycolide Acid) (PLGA)

PLGA is a copolymer approved by the Food and Drug Administration (FDA) for use in therapeutic devices. PLGA is synthesised by ring-opening copolymerisation of two different monomers, namely, cyclic dimers (1,4-dioxane-2,5-diones) of glycolic acid and lactic acid [117]. PLGA is non-toxic as it can decompose into non-toxic products (water and carbon dioxide), which are eliminated from the human body. Overall, PLGA NPs are considered as one of the most versatile drug delivery systems in the field of nanomedicine due to its numerous advantages such as biodegradability, biocompatibility, high stability, no toxicity, and a controlled degradation rate that depends on the molecular weight of PLGA and the ratios of poly-glycolic acid and poly-lactide acid. A variety of methods have been used to synthesise PLGA NPs, including emulsification, nanoprecipitation, dialysis, and spray drying [118]. The intrinsic properties of PLGA make it suitable for the encapsulation and delivery of small molecule drugs, such as flavonoids and other natural active small molecules [119]. For example, Wu et al. established a PLGA-based nano-delivery system loaded with EGCG and found that PLGA could improve the stability of EGCG by preventing its oxidation, thereby significantly increasing its bioactivity [120]. In addition, the surface modification on PLGA also plays a key role in targeting strategy, retention time, and biocompatibility. For example, when PLGA is bound to other polymers, such as polyethylene glycol (PEG) and polyvinyl alcohol (PVA), the retention time of PLGA NPs, as well as the blood half-life, appears to increase. Chen et al. modified the surface of PLGA NPs with biotinylated chitosan and found that the cytotoxicity of epirubicin was apparently increased [121]. 

#### 3.3.2. Poly(ε-Caprolactone) (PCL)

Poly(ε-caprolactone) (PCL) is another synthetic polymer approved by the FDA for use in drug delivery devices. PCL is produced by ring-opening polymerisation of ε-caprolactone using a catalyst and can be biodegraded by lipase. PCL is a semi-crystalline biodegradable polymer with hydrophobicity, which has high biodegradability and drug binding capacity [122]. In addition, PCL is colloidally stable in biological fluids, easily crosses cell membranes by endocytosis, has low toxicity in vitro and in vivo, and provides controlled drug release [123]. For these reasons, PCL has been widely used in drug delivery systems. For example, PCL-encapsulated quercetin-biapigenin nanoparticles are able to effectively penetrate the blood-brain barrier [124].

#### 3.3.3. Dendrigraft Poly-L-Lysine (DGL)

The polycondensation of a lysine forms a poly-L-lysine dendrigraft (DGL), which is a water-soluble polycationic dendrigraft based on poly-L-lysine. With a linear core, the structure of DGL is more flexible than that of dendrigrafts. As a synthetic polymer, DGL is biodegradable, monodispersible, and biofilm permeable, and it can be combined with anionic groups [125]. DGL is degradable to amino acid, which is less toxic. In addition, the molecular surface of DGL has a large number of amino molecular groups, and more group modifications occur in the complex molecular structure. The shape of DGL is generally spherical and positively charged with electricity [126]. Recently, DGL and its derivatives have been increasingly investigated in nano-delivery systems. For example, peptide-guided DGL nanoparticles are able to deliver prodigiosin to choriocarcinoma, making them an excellent targeted delivery vehicle [127].

#### 3.3.4. Polyanhydride

As a synthetic polymer, polyanhydride is biodegradable, non-toxic, biocompatible, and has sustained release and surface erosion properties. They can be manufactured at low cost from available resources and designed to meet the required properties. Polyanhydrides are degraded in vivo to non-toxic diacid counterparts, which are eliminated from the human body as metabolites [128]. Currently, polyanhydrides are considered to be useful synthetic biomaterials as drug carriers. For example, due to their effective modulation of active proteins released in vivo, polyanhydride NPs are able to induce low-level inflammatory activation of dendritic cells, leading to CD8+ T cell memory and delayed cancer development [129].

**Table 1:** Biodegradable polymers: sources, merits, and demerits.

Biodegradable Polymers	Name	Sources	Merits	Demerits
Natural biodegradable polymers	Chitosan	Exoskeleton of crustaceans	Biodegradability, low cost biocompatibility, mucosal immune [103].	Soluble in acidic solutions, limited application [130].
	Zein	Corn	Biodegradability, low toxicity, biocompatibility, low cost [107].	Soluble in water containing organic solvents, limited application [106].
	Alginate	Algae	Low toxicity, low cost, used in mucous membranes and traverse body [111].	Tedious preparation process, no targeting [131].
	Hyaluronic acid	Animal tissue, microbial	Biocompatibility, low toxicity [132].	Mass-production may lead to impurity, high price of biological extraction [133].
Synthetic biodegradable polymers	Poly(lactide-co- glycolide acid)	Polymerization of lactic acid and glycolic acid	Loading multiple antigens and immune modulators, used in mucous membranes and traverse body [134].	Organic solvents are required, lack of stability, mucosal administration is ineffective [135].
	Poly (ε-caprolactone)	Polymerization of ε-caprolactone	Biodegradability, colloidal stable, low toxicity, facile celluar uptake [122].	Slow degradation rate, poor mechanical properties, low cell adhesion [123].
	Dendrigraft poly-L-lysine	Lysine polycondensation synthesis	Low toxicity, targeted [136].	Preparation requires complex coupling processes, immunogenicity may interfere with booster immunity [125].
	Polyanhydride	Methyl vinyl ether-maleic hydride synthesis	Sustained release, surface erosion [137].	Highly sensitive to hydrolysis, limited application [138].

### 3.4. Delivery Routes of Biodegradable Polymeric NPs in IBD

Lai et al. used muco-inert polymers to prepare NPs as large as 500 nm and found that they were able to rapidly penetrate physiological human mucus, with diffusivities up to four times slower than those in pure water [139]. Thus, NPs are capable of penetrating the mucus layer and delivering loaded drugs directly to intestinal cells. They can also be taken up by macrophage cells via phagocytosis, allowing modulation of the immune environment in the gut [140]. Surface modifications allow NPs to adhere to target tissues for prolonged periods, making them useful for therapeutic applications [141]. Through the above-mentioned pathways, polymeric NPs can greatly enhance the efficacy of flavonoids in IBD. Currently, the main delivery methods of polymeric NPs in IBD include oral administration, injection, and rectal administration. The flavonoid-based therapy of IBD is mainly through oral administration, which is more convenient and has higher patient compliance. Therefore, we will focus on the oral route of polymeric NPs in IBD.

#### 3.4.1. pH-Sensitive Polymeric NPs

There are dramatic differences in pH between different regions of the gastrointestinal tract. Therapeutic agents that are not protected from pH variations can lose their bioactivity through processes such as hydrolysis, oxidation, and deamination [142]. The pH sensitivity of polymeric NPs allows them to protect their payload as they pass through the initial acidic environment of the stomach, and then release the drugs once they reach a pH environment that is favourable for dissolution or swelling. As a result, the use of pH-sensitive polymeric NPs can deliver therapeutic drugs to the specific target tissue in the gastrointestinal tract. 

Eudragit S100 is a pH-sensitive polymer for colon-targeted drug delivery due to its dissolution at pH 7, which has been applied to NPs to facilitate drug release when reaching the colonic lumen at pH 7 [143]. H. Ali et al. prepared plain PLGA-based NPs and PH-sensitive PLGA/Eudragit S100-based NPs, both loaded with budesonide, and then treated various acute and chronic colitis mouse models. In addition, the protective effects of the NPs were assessed and compared with free budesonide. They found that PLGA/Eudragit S100-based NPs reduced intestinal inflammation better than plain PLGA-based NPs, which were more effective than free budesonide. Furthermore, PLGA/Eudragit S100-based NPs were found in higher amounts at the site of inflammation than plain PLGA-based NPs, and both were abundant in the inflamed region of the colon. In contrast, NPs were distributed throughout the gastrointestinal tract. These results support the hypothesis that NPs may accumulate to a greater extent at the site of inflammation [144].

#### 3.4.2. ROS-Responsive Polymeric NPs

UC is often associated with excessive production of ROS. Biopsy samples from UC patients showed dramatic increases in mucosal ROS levels, ranging from 10 to 100 times normal levels [145]. Given the negative effects of ROS-mediated oxidative stress in IBD, ROS has become a target of interest for drug delivery, particularly with the aim of targeting drugs to inflamed tissue. For example, Huang et al. developed ROS-responsive NPs composed of Pluronic F127 (PF127)/PLGA and loaded with curcumin (CUR) and catalase (CAT), termed P-CAT/CUR NPs, and evaluated their protection in a DSS-induced UC mouse model. The results showed that the drug release rate of these NPs increased significantly with increasing H2O2 levels, demonstrating their sensitivity to H2O2. Furthermore, the addition of PF127 to the NPs improved not only their ability to penetrate intestinal mucus, but also their uptake by macrophages, anti-inflammatory effects, and antioxidant capabilities. After oral administration, P-CAT/CUR NPs were found to accumulate effectively in colitis tissue and resulted in superior therapeutic outcomes compared to their control counterparts (CUR NPs and P-CUR NPs) [146].

#### 3.4.3. Targeted Polymeric NPs

Targeted polymeric NPs hold great promise for the treatment and prevention of IBD [147]. By incorporating targeting ligands on their surface, these NPs can effectively accumulate at inflamed sites and allow efficient internalisation by target cells. This targeted approach enhances therapeutic efficacy and minimises the potential for adverse drug effects [148]. Intestinal tissue-resident macrophages, which play a critical role in the development and progression of IBD, have been identified as key target cells in traditional treatment strategies. Macrophages are responsible for defence against foreign pathogens, repair of the intestinal epithelial layer, and regulation of the immune microenvironment [149]. In addition to macrophages, intestinal epithelial cells are also considered key targets in IBD therapy, as the main goals of IBD treatment are not only to reduce inflammation but also to promote mucosal healing [150]. For example, Zhang et al. investigated the targeting potential of galactose to activated macrophages, which highly overexpress galactose receptors under inflammatory conditions. These receptors can facilitate receptor-mediated endocytosis. The team synthesised galactosylated trimethylchitosan cysteine (GTC) and combined it with siRNA against mitogen-activated protein kinase kinase kinase 4 (Map4k4) in the presence of sodium triphosphate (TPP). The results showed that the GTC/TPP/siRNA NPs achieved significantly higher cellular uptake than trimethylchitosan-cysteine (TC)/TPP/siRNA NPs without galactose groups. In vitro and in vivo studies confirmed that TNF-a expression was significantly lower in the GTC/TPP/siRNA NP-treated group compared to the TC/TPP/siRNA NP-treated group. Furthermore, oral administration of the GTC/TPP/Map4k4 siRNA NPs had better therapeutic effects against DSS-induced UC as demonstrated by body weight, colon length shortening, MPO activity, and colon histology [151]. 

#### 3.4.4. Multi-Responsive Polymeric NPs

While some single-stimulus delivery systems have shown promising results, there are still significant concerns regarding the variability of the gut environment in patients at different stages of IBD [152]. These single-stimulus-based formulations may not be sufficiently targeted. To address this issue, scientists have recently attempted to use multi-responsive NPs to achieve more effective targeting [153].

## 4. Applications of Biodegradable Polymeric Nanoparticles in IBD

To date, over 5000 flavonoids have been identified, most of which are beneficial for gut health [21]. As mentioned above, flavonoids may regulate IBD through multiple pathways. However, only a few flavonoid drugs have entered clinical use due to their low bioavailability. With the rapid development of nanodelivery technology, especially the great progress in biodegradable polymers, more and more research has been focused on improving the bioavailability of flavonoids by polymeric nanocarrier technology. In recent years, there have been a large number of studies published online related to the application of various flavonoids encapsulated by different polymers in IBD therapy. Here, we will discuss some flavonoid NPs used in IBD treatment. Details can be found in Table 2.

### 4.1. Quercetin Nanoparticles

Quercetin is a pentahydroxyflavone polyphenol found in olive oil, lettuce, onions, tea, coffee, red grapes, and citrus fruits. As one of the most abundant flavonoids in the diet, quercetin has many health benefits, such as antioxidant and anti-inflammatory properties, antiviral, and anti-tumour properties [164]. In addition, quercetin is relatively safe for the human body when consumed in doses of less than 1000 mg per day [165]. Because of its antioxidant and anti-inflammatory properties, quercetin, like most flavonoids, is able to treat IBD through the protective mechanisms mentioned above. For example, dietary quercetin ameliorated inflammatory levels in DSS-induced colitis mice by regulating the ERK1/2-FKBP and RXR-STAT3 pathways, which are involved in the pathogenesis of IBD [166]. Furthermore, quercetin was able to restore the diversity of the colonic microbiota by increasing the populations of beneficial bacteria and reducing the levels of harmful bacteria, thereby reducing the severity of colitis in Citrobacter rodentium-infected mice [167]. Nevertheless, the clinical efficacy of quercetin in IBD is unsatisfactory due to its low bioavailability.

Recently, some scientists have used polymers to prepare quercetin NPs, apparently improving their bioavailability and therapeutic effect in IBD. For example, Diez-Echave et al. established DSS-induced colitis mice, evaluated the anti-colitis effects of quercetin loaded in silk fibroin nanoparticles, and found that daily administration of these NPs significantly reduced disease activity index values compared to the control colitis group. The protective effect was demonstrated by histological examination and the reduction of several proinflammatory cytokines in colonic tissue [154]. Khater et al. used chitosan NPs to load quercetin and investigated the anti-colitis effects of different concentrations of quercetin NPs on DSS-induced colitis rats. The results showed that administration of quercetin NPs, especially at higher concentrations, significantly decreased the disease activity index and the amount of faecal calprotectin marker compared to the control colitis group. In addition, higher concentrations of quercetin NPs appeared to downregulate proinflammatory cytokines, upregulate genes expressing tight junction proteins, and restore the damaged structures of colonic tissues [155]. Unfortunately, the two studies above did not compare the protective effect of quercetin NPs against IBD with that of free quercetin. However, these data suggest that biodegradable polymeric NPs loaded with quercetin could be a promising alternative to current IBD therapies. Furthermore, Shen et al. designed a novel responsive quercetin-conjugated glycol chitosan prodrug micelles as an inflammation-targeted drug. The optimised micelles showed a low release rate and sustained release property under physiological conditions, and, moreover, they tended to accumulate in inflammatory sites of the colon and showed better therapeutic efficacy than free quercetin in colitis mice. This work improved the inflammation-targeted delivery and accumulation of quercetin in the colon, ultimately enhancing the anti-colitis effect. As a result, the bioactivity of quercetin in vivo is significantly enhanced [156].

### 4.2. Apigenin Nanoparticles

Apigenin is a low molecular weight 4′,5,7-trihydroxyflavone found in most fruits and vegetables, such as parsley, celery, and celeriac, and also in chamomile tea. Apigenin is particularly abundant in the flowers of chamomile, accounting for 68% of total flavonoids [168]. Apigenin is beneficial to human health, particularly for its antioxidant, anti-hyperglycaemic, anti-inflammatory, and anti-apoptotic properties. In addition, apigenin can inhibit various tumour cells [169]. As a dietary flavonoid, apigenin is relatively safe and no toxicity has been reported. However, at high doses, apigenin can cause muscle relaxation and sedation [170]. Like quercetin, apigenin may protect against IBD. For example, Ganjare et al. investigated the protective effect of apigenin on DSS-induced colitis in mice and found that oral administration of apigenin had a marked anti-inflammatory effect associated with inhibition of the NLRP3 inflammatory pathway [171].

Although apigenin has potent anti-inflammatory activity, poor absorption and low availability limit its wider use in IBD. Lv et al. designed novel apigenin-loaded NPs, namely, apigenin-Mn(Ⅱ) complex was loaded into sodium hyaluronate NPs, and evaluated the therapeutic effect of these NPs on DSS-induced colitis mice. They found that the novel apigenin NPs could significantly restore the colonic epithelial barrier and apparently improve the damaged colonic tissue by regulating inflammatory factors. In addition, the apigenin NPs showed better solubility and bioavailability compared to free apigenin. The experimental results also suggested that apigenin NPs produced a stronger anti-colitis effect than free apigenin, apigenin-Mn(Ⅱ) complex, and 5-aminosalicylic acid (5-ASA) [157]. This work is a successful attempt, although only a small number of studies have addressed the application of apigenin nanoparticles in IBD.

### 4.3. Epigallocatechin Gallate (EGCG) Nanoparticles

Epigallocatechin gallate (EGCG), also known as epigallocatechin-3-gallate, is a type of catechin and the ester of epigallocatechin and gallic acid. EGCG is the most abundant catechin in tea, with high levels found in green, white, and black tea. Studies suggest that excessive intake of EGCG, especially above 800 mg daily, may cause liver toxicity [172]. EGCG has many health benefits, such as anti-inflammatory, anti-tumour, cardioprotective, cognitive enhancement, neurodegenerative disease prevention, and carbohydrate metabolism regulation [173]. Recently, scientists have used EGCG to treat IBD in mice. For example, Wu et al. established DSS-induced colitis in mice and evaluated the beneficial effect of EGCG in alleviating IBD. The experimental data suggested that oral EGCG ameliorated the inflammatory level in colitis mice by modulating the intestinal microbiota [28].

Similar to quercetin and apigenin, EGCG has poor intestinal absorption and instability, which limits its use in prevention and therapy. However, encapsulation of EGCG in nanoparticles can improve its stability and therapeutic effects. For example, Dube et al. used chitosan NPs to enhance the intestinal absorption of EGCG in vivo. Furthermore, the same authors found that chitosan NPs improved the plasma exposure of EGCG in mice by enhancing intestinal stability [158,159]. Li et al. used ovalbumin-dextran to prepare nanocarriers with the aim of improving the absorption of loaded EGCG and its stability in the gastrointestinal tract. The experimental data showed that EGCG-loaded ovalbumin-dextran NPs were more stable in simulated gastric and intestinal fluids, and the absorption of EGCG through Caco-2 monolayer models was more enhanced compared to free EGCG [160]. Furthermore, Liu et al. designed a novel EGCG-loaded silk fibroin nanoparticle with surface functionalisation of antimicrobial peptides, and found that these EGCG NPs efficiently repaired the epithelial barrier in the colon by reducing oxidative stress and improving epithelial migration. Meanwhile, they downregulated pro-inflammatory factors and upregulated anti-inflammatory factors. In addition, hydrogel (chitosan/alginate) encapsulating the above NPs could not only treat colitis, but also regulate gut microbiota by enhancing the diversity and richness of intestinal flora and increasing the amount of beneficial bacteria [161].

### 4.4. Naringenin Nanoparticles

Naringenin is a type of flavonoid, which is tasteless and colourless, found in fruits and herbs such as grapefruit, sour orange, tart cherries, tomatoes, water mint, and bergamot, beans. The structure of naringenin is a flavanone with three hydroxyl units on carbons 4′, 5 and 7. Naringenin is found in the aglycol form. In contrast, naringin is its glycosidic form [174]. Ingestion of doses of less than 900 mg of naringenin is relatively safe in healthy individuals [175]. Naringenin has beneficial effects on human health, such as antibacterial, antifungal, antiviral, anti-inflammatory, antioxidant and anticancer properties [176]. In addition, naringenin can be used to treat IBD, as investigated by Chaen et al. They found that dietary naringenin could repair colonic damage in colitis mice by downregulating the expression of epithelial tumour necrosis factor-α (TNF-α) and inducing M2-type macrophages in colitis mice [177].

To improve the poor bioavailability of naringenin, nanomaterials have recently been used to deliver naringenin. For example, Kumar et al. developed novel naringenin NPs using polyvinylpyrrolidone (PVP) as a hydrophilic carrier. They found that PVP-naringenin NPs were safe, reduced the dose of naringenin, and increased its bioavailability through in vivo experiments. Finally, they suggested that these novel NPs are suitable for further applications in disease treatment [162]. Song et al. encapsulated naringenin in a micelle by mixing Pluronic F127 with Tween 80 and evaluated its bioavailability in Sprague-Dawley (SD) rats, finding an apparent increase in naringenin bioavailability (26.9% after oral administration), solubility (27-fold), and intestinal permeability (1.7-fold) compared to free naringenin [163]. Unfortunately, few studies have been reported on the use of naringenin NPs in IBD. 

### 4.5. Synergic Effects of Multiple Flavonoids Nanoparticles

**Figure 6:**
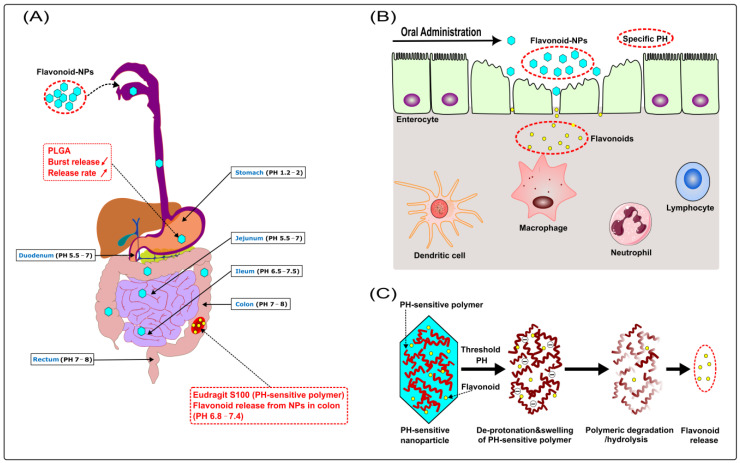
(**A**) PLGA (a biodegradable polymer) reduces the burst release of NPs, whose release rate is also prolonged; Eudragit S100 (a pH-sensitive polymer) protects NPs from destruction in the stomach and small intestine, then releases the flavonoid in the colon (pH ≥ 6.8). (**B**) Disturbed mucosal barrier, loss of epithelial integrity, widening of gaps between enterocytes, and increased recruitment of immune cells are usually present in IBD. The pathological changes disrupt the barrier function of the epithelium with increased translocation of bacteria and loss of fluid and electrolytes. Meanwhile, they facilitate increased accumulation and penetration of NPs across the epithelium and uptake by enterocytes and immune cells such as macrophages. (**C**) Mechanism of flavonoid release from pH-sensitive NPs: Eudragit S100 (a pH-sensitive polymer) deprotonates when exposed to a threshold pH ≥ 6.8, causing polymer swelling, dissolution, and erosion with subsequent flavonoid release. (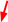 decrease; 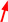 increase).

Recently, Zhang et al. proposed that combined phytochemicals exert synergistic anti-inflammatory functions by improving the uptake/bioavailability of each phytochemical through enhancing antioxidant properties, interacting with gut microbiota, and targeting the same cells/signaling pathways as well as different cells/signaling pathways [178]. For example, combined administration of genistein and EGCG improved EGCG levels in the small intestine [179]. Furthermore, a combination treatment of quercetin and resveratrol produced a synergistic anti-inflammatory effect in rats fed a high-fat sucrose diet [180]. Furthermore, Mitra et al. reviewed 15 types of polyphenols from a variety of studies, such as kaempferol, quercetin, myricetin, cyanidin, morin, apigenin, and genistein. The 15 types of polyphenols have potential synergistic effects, including anti-inflammatory effects. The other polyphenols had different effects but no apparent synergism [181]. For example, a combination treatment of quercetin and catechin synergistically produced anti-inflammatory effects on lipopolysaccharide (LPS)-stimulated RAW 264.7 macrophage cells [182]. For these reasons, it is conceivable that a combination of several flavonoids may also have synergistic anti-inflammatory effects in IBD. However, few studies have focused on the synergistic anti-inflammatory effects of multiple flavonoids in IBD. In a preliminary experiment, our group found that oral administration of a flavonoid combo, mainly consisting of apigenin and EGCG, was able to significantly ameliorate the inflammatory level of oxazolone-induced colitis in mice. Unfortunately, we have not yet compared the flavonoid combination with a single component. For the same principle, there’s reason to believe that complementary synergistic effects of multiple flavonoid NPs should also occur in the prophylaxis and treatment of IBD. Currently, our group is preparing multiple flavonoid PH-sensitive NPs composed of PLGA and Eudragit S100, including quercetin, apigenin, EGCG, and naringenin (for detailed mechanisms, see Figure 6). Subsequently, we plan to evaluate the protective effect of flavonoid NPs combination as well as single component NPs on IBD, and simultaneously perform a comparative analysis (data not shown). Further studies are needed to evaluate this.

## 5. Challenge and Perspective

Since flavonoids are easily oxidised, they are not stable and degrade rapidly in vivo and in vitro. However, through the versatile structures of polymers, polymeric NPs can be designed and produced in various formulations with the required properties, allowing flavonoids to be encapsulated by polymers and avoid rapid degradation. Yadav et al. prepared polymeric NPs loaded with curcumin that were stable for up to 6 months of storage at 2–8 °C and ambient conditions [183]. Therefore, high stability is a major advantage of polymeric NPs. Currently, there are still some challenges that limit the clinical application of flavonoid-loaded NPs, such as concerns about long-term biological safety, limitations of characterisation methods, and obstacles to large-scale production [184]. On the one hand, although polymeric NPs are biodegradable, several factors (surface charge, particle size, shape and porosity, as well as biodegradability) can cause the toxicity of NPs. In addition, it is difficult to assess the toxicity of NPs due to the batch-to-batch variations that often occur in large-scale production [185]. On the other hand, current production techniques hardly eliminate all the organic solvents used in the production process of NPs [186]. These residual solvents have the potential to cause adverse effects such as neurotoxicity, anaemia, and leucopenia [187]. Furthermore, the harsh environment of the gastrointestinal tract is considered to be another challenge in drug delivery systems, which may prevent drugs from reaching inflamed tissues. Drugs or their NPs can only have satisfactory therapeutic effects if the drugs are delivered precisely to the focal tissues [188]. As a result, it’s difficult to achieve targeted drug delivery to focal tissues, target cells and specific organs. To date, there is limited clinical data on the use of polymeric NPs for the treatment of IBD, particularly flavonoid-loaded polymeric NPs, which have not been widely used in the clinic. Consequently, there is a lack of evidence and data on the adverse effects of polymeric NPs.

Flavonoids have many advantages over synthetic drugs due to their versatile structures and target diversity. Further research on flavonoids will lead to the confirmation of new therapeutic targets and new signalling pathways against IBD. Although polymeric NPs enhance the therapeutic effects of flavonoids in IBD, very few NP formulations have entered clinical trials. To accelerate the clinical translation of flavonoid-loaded polymeric NPs, some improvements should be made. For example, solvent-free or solventless technologies should be used in the preparation of NPs [189]. Second, polymeric NPs, which are biocompatible, biodegradable, inexpensive, and easy to batch produce should be the focus of research. Thirdly, more research should be devoted to solving practical problems: the toxicity-metabolism characterisation pathways and various production techniques of polymeric NPs may be of great interest in the future. All these measures will accelerate the clinical translation of flavonoid-loaded NPs to some extent in the future.

## 6. Conclusions

Inflammatory bowel disease is an incurable disease. Its aetiology remains unclear. Today, all drugs and procedures on the market are only able to induce and maintain remission in IBD patients, not to bring about a complete cure. Conventional drugs (5-ASA, corticosteroids), immunosuppressants (methotrexate, cyclosporine A, tacrolimus), and biologics (anti-TNF-α antibodies) have proven to be effective anti-inflammatory agents, but most of them are not specific enough and also cause systemic side effects. As an alternative, EGCG was first used as a pilot study in the treatment of IBD due to its safety and multiple anti-inflammatory effects. However, the low bioavailability of flavonoids from food severely limits their use in clinical treatment, and for this reason nanoparticulate transporters are desirable for its supply. In recent years, especially, scientists have attempted to produce biodegradable polymeric nanoparticles loaded with various flavonoids to increase their bioavailability or bioactivity in vivo and improve their therapeutic effect. 

So far, one clinical trial has shown that EGCG may be suitable for the treatment of IBD [29]. However, little is known about the use of flavonoid-loaded NPs in the clinic for the treatment of IBD. Now, with the help of some preclinical models, it has been shown that flavonoid-loaded NPs could greatly improve the therapeutic effect of flavonoids in IBD. Consequently, there are reasons to believe that flavonoid-loaded polymeric NPs may have great potential for the treatment of IBD in the clinic. With the conduct of further clinical trials, flavonoid-loaded polymeric NPs have a high probability of becoming a successful therapeutic option for the treatment of IBD in the future.

## Figures and Tables

**Table 2:** Flavonoids loading in nanoparticles for IBD treatment.

Flavonoid	Nanometerial	Model	Effects	Reference
Quercetin	Silk fibroin	DSS mice	Reduced disease activity index, histological damage and proinflammatory cytokines.	[154]
	Chitosan	DSS rats	Reduced disease activity index, fecal calprotectin marker and proinflammatory cytokines, upregulated genes expressing tight junction proteins, prevented mucosal damage.	[155]
	Conjugated glycol chitosan prodrug micelles	DSS mice	Better therapeutic efficacy than free quercetin.	[156]
Apigenin	Sodium hyaluronate	DSS mice	More powerful anti-colitis effect, higher solubility and bioavailability compared with free apigenin.	[157]
EGCG	Chitosan	Excised mouse jejunum	Enhanced intestinal absorption of EGCG.	[158]
	Chitosan	Mice	Improved plasma exposure of EGCG by enhancing the intestinal stability.	[159]
	Ovalbumin-dextran	Caco-2 cells	More stable and higher absorbance than free EGCG.	[160]
	Silk fibroin, surface functionalization of antimicrobial peptides, hydrogel (chitosan/alginate)	DSS mice	Repaired epithelial barrier, downregulated proinflammatory factors, upregulated anti-inflammatory factors, regulated gut microbiota.	[161]
Naringenin	PVP	SD rats	Increased bioavailability.	[162]
	Mixed micelle of Pluronic F127 and Tween 80	SD rats	Increased bioavailability, solubility and intestinal permeability.	[163]

## Data Availability

Not applicable.
